# Great saphenous vein leiomyosarcoma: a rare case report and literature review

**DOI:** 10.3389/fonc.2025.1589001

**Published:** 2025-07-02

**Authors:** Ying Liu, Bin Dong, Chunjie Hou, Litao Sun, Genshan He, Qiaohong Hu

**Affiliations:** ^1^ Cancer Center, Department of Ultrasound Medicine, Zhejiang Provincial People’s Hospital (Affiliated People’s Hospital), Hangzhou Medical College, Hangzhou, Zhejiang, China; ^2^ Department of Ultrasonography, Hangzhou Third People’s Hospital & Hangzhou Third Hospital Affiliated to Zhejiang Chinese Medical University, Hangzhou, China

**Keywords:** leiomyosarcoma, great saphenous vein, ultrasound, hypoecho, case report

## Abstract

Great saphenous vein leiomyosarcoma is an extremely rare tumor clinically, which is often misdiagnosed as superficial venous thrombosis due to atypical clinical manifestations. This report describes a rare presentation of great saphenous vein leiomyosarcoma in a 37-year-old man who was referred to our hospital due to a gradually enlarging painless mass on his left shank. Ultrasound revealed that the mass in the left calf was a solid tumor originating from the left great saphenous vein. He underwent radical resection for the mass ultimately. Subsequent histopathological and immunohistochemical analyses resulted in a conclusive diagnosis of primary leiomyosarcoma originating from the great saphenous vein. There are only 56 cases of great saphenous vein leiomyosarcoma reported in medical databases worldwide at present, which are summarized and analyzed here.

## Introduction

Leiomyosarcoma (LMS) is a rare malignant tumor, and LMS originating from the vessel is even rarer (1). Vascular LMS usually occurs in the inferior vena cava and is relatively rare in the great saphenous vein (GSV) (2). Herein, we report a case of leiomyosarcoma of the great saphenous vein (GSV-LMS) and review the literature. The patient provided written informed consent for the publication of this manuscript and any identifying images or data. The clinical manifestations of GSV-LMS are atypical (3), and only 56 cases have been publicly reported so far. There is a lack of experience in its diagnosis and treatment, which leads to a lack of understanding. As a consequence, this disease is prone to misdiagnosis and mistreatment. By means of the introduction of a rare case, we analyzed the diagnostic value of GSV-LMS based on ultrasound, combined with the contents of previous literature, and we tried to summarize the effective diagnosis and treatment information of GSV-LMS so as to improve the preoperative diagnostic accuracy and the prognosis of patients.

## Case description

### Case presentation

A previously healthy 37-year-old man was admitted to our hospital due to a 4-month history of a subcutaneous mass about the size of a walnut on his left calf. He did not take it seriously at first. The patient reported that the mass gradually increased and was currently about the size of an egg. In the past week, the skin of the lump was accidentally broken. Prior to hospitalization, he had been undergoing symptomatic treatment with no obvious improvement. On the first day of admission, the patient’s physical examination revealed that there was a 6 cm × 4 cm mass in the inner part of the lower left calf. It was hard, slightly compressible, with low mobility and no obvious tenderness. The skin tension on the surface was high, accompanied by pigmentation, local skin rupture, and a small amount of serous exudates.

### Ultrasound findings

**Figure 1:**
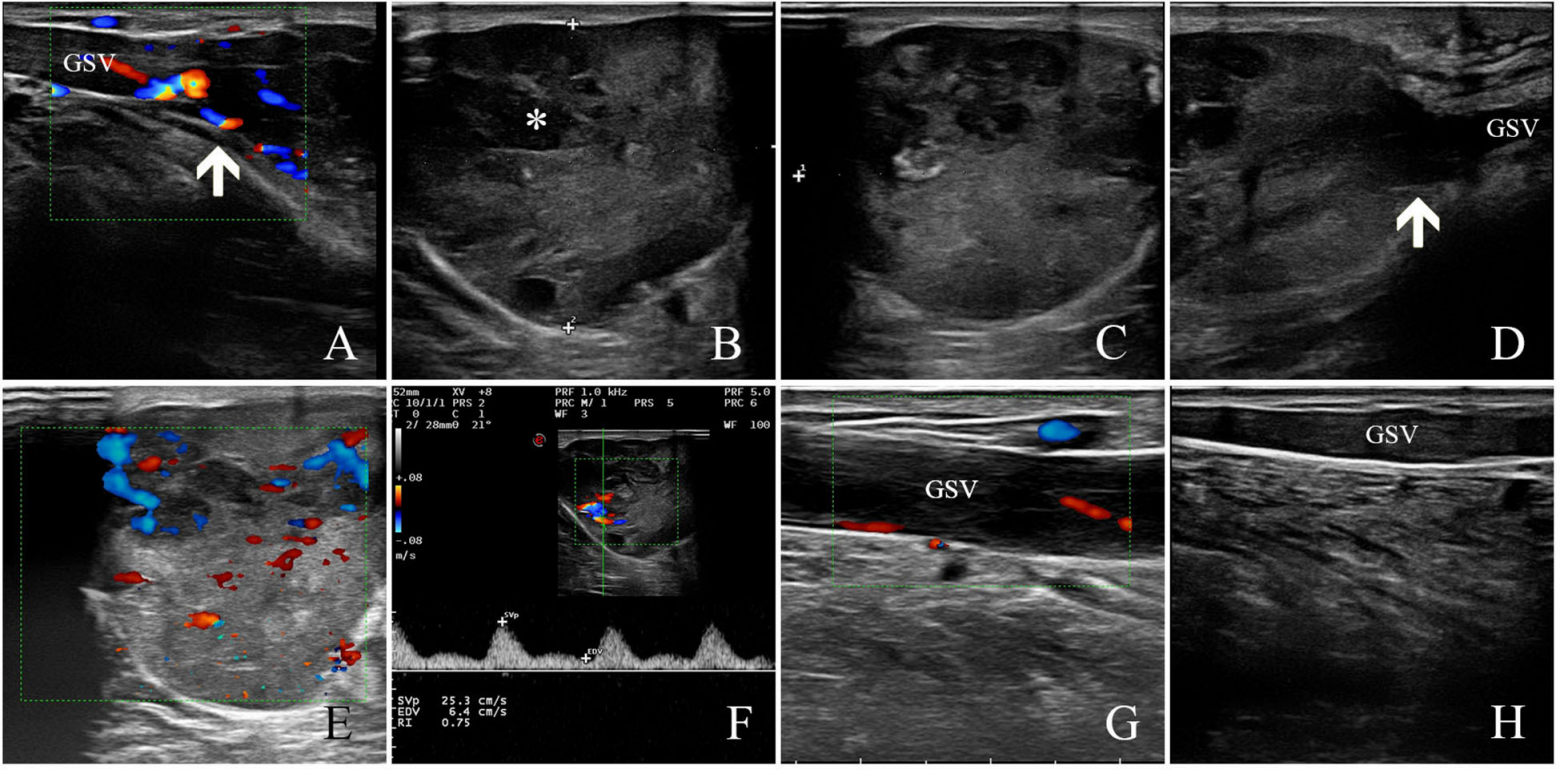
Ultrasound findings of GSV-LMS in a male patient. **(A)** The white arrow indicates the upper pole of the lesion. **(B, C)** The figure shows that the internal echo was heterogeneous hypoechoic, and a number of honeycomb-like anechoic areas were detected (as shown by white * in the figure). **(D)** The white arrow indicates the lower pole of the lesion. **(E)** Color Doppler flow imaging (CDFI) showed abundant blood flow signals in the mass. **(F)** The arterial spectrum was detected in the internal blood flow signal of the lesion with a resistance index of approximately 0.75. **(G)** The GSV at the proximal side of the lesion was full of heterogeneous hypoechoic mass, and a punctured blood flow signal could be detected. **(H)** The GSV at the distal side of the lesion was full of heterogeneous hypoechoic mass, and no blood flow signal could be detected. GSV, great saphenous vein; LMS, leiomyosarcoma.

Ultrasonography showed a subcutaneous heterogeneous hypoechoic mass located in the inside of his lower left calf, the size of which was approximately 5.9 cm × 3.6 cm × 4.5 cm, the shape was lobed, the boundary was clear, and the edge was not smooth (**Figures 1A–D**). The interior of the mass was detected to have a number of honeycomb-like anechoic areas (**Figures 1B, C**). Ultrasound scan showed that the upper and lower poles of the mass connected to the GSV (**Figures 1A, D**), and the residual GSV of the left calf had a wide diameter and was filled with a heterogeneous hypoechoic material (**Figures 1G, H**). Color Doppler flow imaging (CDFI) showed abundant blood flow signals in the mass and punctate blood flow signals in the heterogeneous hypoechoic mass in the residual GSV of the left calf (**Figures 1E–G**). In addition, there was no obvious abnormality in the inferior vena cava, iliac vein, and other deep or superficial veins of the lower limbs, and the blood flow signal was normal. The ultrasound diagnosis was a solid tumor originating from the GSV. The patient has a history of diabetes for more than 1 year with regular medication and a satisfactory level of control. The patient’s chest radiograph, electrocardiogram, and other preoperative routine laboratory tests were normal.

### Treatment

**Figure 2:**
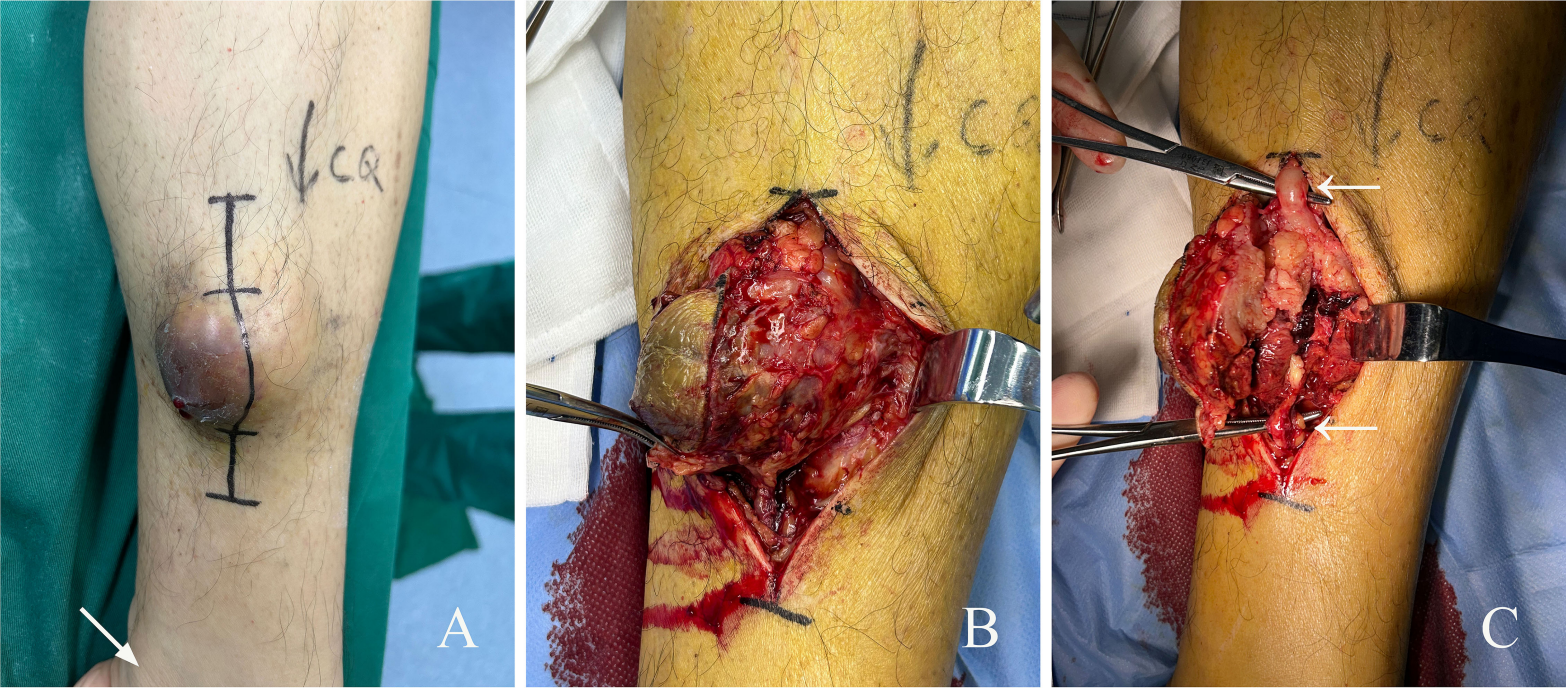
Surgical findings of GSV-LMS in a male patient. **(A)** Appearance of the subcutaneous mass on his left calf. The area indicated by the arrow is the medial malleolus. **(B)** Intraoperative view showing a mass with rich blood supply approximately 6 cm × 7 cm × 5 cm in size was found wrapped around the GSV. More proliferative vessels can be seen on the surface and around the mass. **(C)** The mass was isolated and confirmed to be of GSV origin. The white arrows mark the GSV. GSV, great saphenous vein; LMS, leiomyosarcoma.

The patient underwent excision of soft tissue mass in his lower left calf under general anesthesia (**Figure 2A**). Intraoperative view showed a mass with rich blood supply approximately 6 cm × 7 cm × 5 cm in size found wrapped around the GSV (**Figure 2B**). The mass was isolated and confirmed to be of GSV origin (**Figure 2C**). Rich blood supply was seen on the surface and surrounding area of the mass (**Figure 2B**). The upper and lower blood vessels that connected with the tumor were separated and cut off at approximately 2 cm of the normal segment, not only to avoid embolization risk but also to prevent tumor invasion. The tumor and surrounding adipose tissue were completely removed. No other adjuvant therapy such as radiotherapy or chemotherapy was given after surgery, and the incision healed well.

### Pathological examination

**Figure 3:**
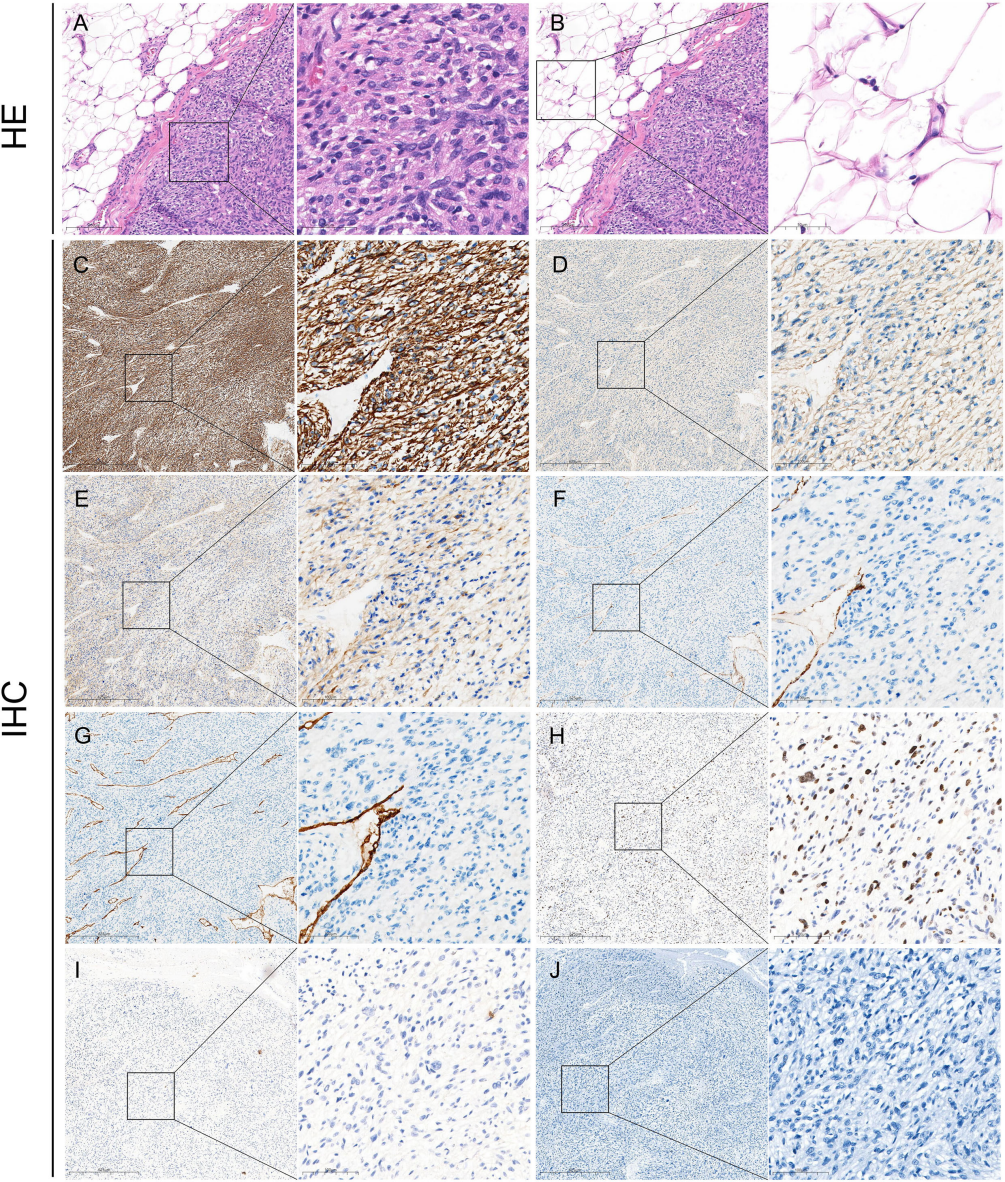
Pathological findings of GSV-LMS in a male patient. **(A)** Left panel: H&E-stained pathological section of this case (×100). Right panel: a locally magnified view (×400) of the area within the box in the left panel showing the venous leiomyosarcoma tissue. It showed fusiform tumor cells with different nuclear sizes, irregular shapes, and large atypia, suggesting that the soft tissue pleomorphic tumor was more likely to be sarcoma. **(B)** Left panel: H&E-stained pathological section of this case (×100), identical to the left panel in panel **(A)** Right panel: a locally magnified view (×400) of the area within the box in the left panel, showing the surrounding lesion tissue. **(C)** IHC staining for Desmin is partially positive. **(D)** IHC staining for SMA is partially positive. **(E)** IHC for h-caldesmon is partially positive. **(F)** IHC staining for CD31 shows positivity only in vascular structures. **(G)** IHC staining for CD34 shows positivity only in vascular structures. **(H)** IHC staining for Ki-67 is positive (30%). **(I)** IHC staining for CK is negative. **(J)** IHC staining for S-100 is negative. GSV, great saphenous vein; LMS, leiomyosarcoma; IHC, immunohistochemistry.

Gross pathology showed that the section of the tumor was grayish-white and grayish-red with medium texture (**Figure 2C**). Microscopic pathology showed fusiform tumor cells with different nuclear sizes, irregular shapes, and large atypia, suggesting that the soft tissue pleomorphic tumor was more likely to be sarcoma (**Figures 3A, B**). Immunohistochemical (IHC) results (**Figures 3C–J**) showed CK (−), SMA (focally +), Desmin (focally +), h-caldesmon (focally +), CD31 (vascular +), CD34 (vascular +), S-100 (−), and Ki-67 (+, 30%). CD31 (vascular +) and CD34 (vascular +) confirmed extensive vascular proliferation within and on the surface of LMS. Ki-67 (+, 30%) supported the diagnosis of high-grade angioleiomyosarcoma.

### Follow-up

The patient has been followed up for approximately 13 months after surgery. Liver metastasis and lung metastasis were found 10 months after operation. The patient received subsequent systemic treatment, including chemotherapy and transarterial chemoembolization (TACE) interventional therapy for liver metastasis. Currently, the patient is alive.

## Literature review

The PubMed, China National Knowledge Infrastructure, Wanfang Data, and Embase databases were searched for articles published from 1868 to 2024. A total of 56 cases in 53 reports were identified: 27 men (48.2%) and 29 women (51.8%). The details are shown in **Table 1**. The age ranged from 2 to 85 years (average 55.64 ± 16.43). Except for six patients whose reasons for visiting the hospital were not reported, 45 patients (90%) were treated for palpable masses, of which 30 masses were painless; two patients (4%) were treated for lower limb edema, and three patients (6%) were treated for superficial phlebitis. The thigh (28 cases, 54.9%) was the most common site of GSV-LMS, followed by the groin (10 cases, 19.6%), shank (nine cases, 17.6%), ankle (three cases, 5.9%), and knee (one case, 2.0%); and no specific site of disease was reported in five cases.

**Table 1:** The 57 cases of great saphenous vein leiomyosarcoma.

Case	Author	Reported year	Reported nation	Gender/age (years)	Site	Initial symptom	Size (mm)	Operation	Adjuvant therapy	Local recurrence	Metastasis	Postoperative follow-up (months)	Outcome
# 1	Aufrecht (4)	1868	Germany	M/23	Ankle	Painful mass	25	Radical resection	No	No	No	NA	NA
# 2	Van Ree (5)	1919	NA	F/42	Left ankle	Edema	NA	Radical resection	No	No	No	15	Alive
# 3	Deweese (6)	1958	USA	M/54	Left thigh	Painful mass, edema	60	Radical resection	NA	NA	NA	NA	NA
# 4	Smout (7)	1960	UK	F/76	Left thigh	Painless mass	40	Extended resection	Radiotherapy	No	No	96	Death
# 5	Dorfman (8)	1963	USA	M/56	Right knee	Painless mass	30	Radical resection	No	No	No	12	Alive
# 6	Christiansen (9)	1964	Denmark	F/64	Left thigh	Painless mass	45	Radical resection	No	No	No	2	Alive
# 7	Allison (10)	1965	UK	F/3	Right groin	Painless mass	25	Extended resection	No	No	No	6	Alive
# 8	Szasz (11)	1969	Canada	M/68	Right thigh	NA	55	Radical resection	No	Yes	Liver/check	48	Death
# 9	Hughes (12)	1973	UK	F/53	Left thigh	Painless mass	25	Radical resection	No	Yes	No	6	Alive
# 10	Jernstrom (13)	1975	USA	M/64	Left groin	Painless mass	120	Radical resection	No	No	No	14	Alive
# 11	Gross (14)	1975	UK	M/46	Right thigh	Painless mass	50	Radical resection	Radiotherapy	No	Thyroid/subcutaneous	36	Alive
# 12	Stringer (15)	1977	USA	M/39	Left thigh	Painful mass	NA	Radical resection	No	No	No	96	Alive
# 13	Stringer (15)	1977	USA	F/36	Left thigh	Painful mass, edema	NA	Radical resection	Radiotherapy and chemotherapy	No	Lung/scalp/chest/bone/heart	132	Death
# 14	Orsi (16)	1979	Italy	M/72	Right thigh	NA	60	Radical resection	NA	NA	NA	NA	NA
# 15	Fischer (17)	1982	USA	F/66	Left groin	Asymptomatic mass	20	Radical resection and femoral vein reconstruction	No	No	No	108	Alive
# 16	Berlin (18)	1984	Sweden	M/60	Right groin	Painless mass, femoral vein thrombosis	30	Extended resection	No	No	Lung/liver	1	Death
# 17	Leu (19)	1986	Switzerland	M/40	Thigh	Small nodule	10	Radical resection	No	No	No	216	Alive
# 18	Humphrey (20)	1987	USA	M/45	Right groin	Asymptomatic mass	25	Radical resection	Radiotherapy	No	No	36	Alive
# 19	Song (21)	1991	Korea	F/54	Left thigh	Painless mass	50	Radical resection	NA	No	No	NA	NA
# 20	Welk (22)	1991	Germany	F/35	NA	NA	50	Radical resection	Radiotherapy	No	No	11	Alive
# 21	Dzsinch (23)	1992	USA	F/70	NA	NA	NA	Radical resection	NA	No	No	204	Alive
# 22	Dzsinch (23)	1992	USA	F/54	NA	NA	NA	Radical resection	NA	No	Lung	9	Alive
# 23	Saglik (24)	1992	Turkey	F/61	Right thigh	Painless mass	60	Radical resection	Chemotherapy	Yes	Lung	66	Alive
# 24	Stallard (25)	1992	USA	F/64	Right groin	Painless mass	70	Radical resection	No	No	No	NA	NA
# 25	Stambuk (26)	1993	Chile	M/48	Right thigh	Painless mass	41	Radical resection	Radiotherapy	No	No	12	Alive
# 26	Byard (27)	1993	USA	F/2	Left thigh	Painless mass	25	Extended resection	Chemotherapy	Yes	No	144	Alive
# 27	Reix (28)	1998	France	M/64	NA	NA	50	Radical resection	Chemotherapy	No	Skin/lung/brain	72	Death
# 28	Zhang (29)	2001	China	M/58	Left shank	Painless mass	NA	NA	NA	NA	NA	NA	NA
# 29	Le Minh (30)	2004	France	F/52	Right thigh	Asymptomatic swelling	20	Extended resection	Radiotherapy and chemotherapy	No	No	12	Alive
# 30	Le Minh (30)	2004	France	M/66	Right thigh	Asymptomatic swelling	50	Radical resection	Radiotherapy	No	No	6	Alive
# 31	van Marle (31)	2004	Netherlands	F/85	Right groin	Painful mass	36	Radical resection	No	No	NA	2	Alive
# 32	Yao (32)	2004	China	F/33	Left groin	thrombosis	170	Radical resection	No	Yes	No	4	Alive
# 33	Zhang (33)	2005	China	F/59	Right thigh	Painful mass	50	Radical resection	Radiotherapy	No	No	4	Alive
# 34	El Khoury (34)	2006	Canada	M/60	Left thigh	Painless mass	30	Radical resection	No	No	No	6	Alive
# 35	Zhang (35)	2006	China	F/59	Right thigh	Painful mass	40 and 60	Radical resection	Radiotherapy	No	No	10	Alive
# 36	Mammano (36)	2008	Italy	M/48	Right groin	Painless mass	60	Radical resection and femoral vein reconstruction	No	No	Lung	30	Death
# 37	Yanada (37)	2010	Japan	M/79	Right thigh	Asymptomatic mass	30	Radical resection	NA	NA	NA	NA	NA
# 38	Bibbo (38)	2011	USA	F/64	Right ankle	Painless mass	70	Radical resection	Radiotherapy	No	No	12	Alive
# 39	Ropcke (39)	2012	Denmark	F/63	Right thigh	Painful mass	NA	Radical resection	NA	NA	NA	NA	NA
# 40	Eris (40)	2012	Turkey	F/67	Above knee	Painful mass	150	Radical resection	No	No	No	24	Alive
# 41	Amato (41)	2013	Italy	M/72	Left thigh	Painless mass	60	Radical resection	Radiotherapy	No	Lung/bone	11	Death
# 42	Cui Xu (42)	2013	China	F/65	Right shank	Painful mass, thrombosis	30	Radical resection	NA	NA	NA	NA	NA
# 43	Werbrouck (43)	2013	Belgium	M/57	Above knee	Painless mass	26	Radical resection	No	NA	NA	NA	NA
# 44	Fremed (44)	2014	USA	M/59	Right thigh	Lower extremity edema, extensive Deep venous thrombosis (DVT)	40	Radical resection and femoral vein reconstruction	NA	No	NA	6	Alive
# 45	Amato (45)	2015	Brazil	M/56	right shank	Painful mass	17 and 19	Radical resection	No	No	No	NA	NA
# 46	Lin (46)	2016	Australia	M/76	Right shank	Painful lump	20	Extended resection	No	No	No	6	Alive
# 47	Cangiano (1)	2017	Italy	F65	Left thigh	Painless mass	30	Radical resection	No	No	No	10	Alive
# 48	Macarenco (47)	2018	Brazil	M/57	Right shank	Painless mass	67	Extended resection	Radiotherapy	No	No	39	Alive
# 49	Naouli (48)	2018	Morocco	M/45	Right thigh	Painless swelling	30	Extended resection	No	No	No	6	Alive
# 50	Zhang (3)	2019	China	M/83	Right shank	Painful mass	40	Radical resection	No	No	NA	6	Alive
# 51	Güner (49)	2020	Turkey	M/34	NA	History of Leiomyosarcoma of the great saphenous vein(GSLMS)	NA	Extended resection	NA	No	Cardiac metastasis	36	Alive
#52	Tresgallo-Parés (50)	2021	Puerto Rico	F/67	Right shank	Painless mass	40	Extended resection	Radiotherapy	No	No	24	Alive
#53	Alkhaled (51)	2022	Saudi Arabia	F/49	Left shank	Painful mass	16	Radical resection	No	No	No	6	Alive
# 54	Fu (52)	2022	China	F/55	Right thigh	Painful mass	100	Radical resection	NA	NA	NA	NA	NA
# 55	Dziekiewicz (53)	2022	Poland	F/61	Below knee	Superficial thrombophlebitis symptoms	100 and 50	Radical resection	No	No	No	12	Alive
# 56	Ammar Atieh (54)	2024	Iran	F/63	Groin	Painless swelling	60	Extended resection	Chemotherapy	No	Lung	NA	Alive
# 57	This case	2024	China	M/37	Left thigh	Painless swelling	60	Radical resection	No	No	Lung/liver	13	Alive

Except for one case with incomplete data, the remaining 55 patients underwent surgical treatment, 41 (74.5%) underwent complete resection, and 11 (20%) underwent wide resection of which three (5.5%) underwent intraoperative revascularization. Intraoperative measurements showed that the maximum diameter of the mass was 10–170 (48.69 ± 32.37) mm. A total of 43 cases (76.8%) were followed up for 1–216 (37.53 ± 52.847) months, only nine cases (20.9%) were followed up for more than 5 years, and seven cases’ follow-up (16.3%) was terminated due to death. Except for nine cases whose postoperative adjuvant therapy was not reported, 18 cases (38.3%) received postoperative adjuvant therapy, including 12 cases who received radiotherapy alone, four cases who received chemotherapy alone, and two cases who received radiotherapy combined with chemotherapy. In addition, except for 11 cases with unknown medical history, five cases (11.1%) had *in situ* recurrence, and 11 cases (24.4%) had distant metastasis. The most common site of metastasis was the lung (eight cases), followed by the liver (two cases), heart (one case), and bone (one case).

## Discussion

Soft tissue sarcomas are rare, accounting for less than 1% of all malignancies, and LMS accounts for only 5%–10% of all soft tissue sarcomas (1, 4). LMS of vascular origin is extremely rare and accounts for less than 2% of all LMS cases. The number of venous origins is five times that of arterial origins (30), and less than 2% of patients had large and middle vein origins (18). The most common site of venous LMS is the inferior vena cava, followed by the renal vein and GSV (2). The GSV is the most commonly involved site in lower limb blood vessels (approximately 30%) (44). However, only 56 cases of GSV-LMS have been reported globally at present, and all of them are case reports and series. The etiology of this disease is unknown, with an insidious onset, difficult diagnosis, and a high susceptibility to misdiagnosis.

GSV-LMS is extremely rare, with only 56 cases reported in the literature (**Table 1**), and our patient is the 57th documented case and the youngest ever reported in China. Moreover, the average age of onset of GSV-LMS is 55.89 ± 17.67 years, and the prevalence is equal between men and women, which was consistent with those previously reported (7). The site of the disease is more common around the knee, while occurrence in the distal end of the knee is relatively rare (3). In this case, the site is the calf, which is an unusual position. The growth patterns of GSV-LMS include intracavitary growth, extracellular growth, and mixed growth (55). The clinical manifestations of GSV-LMS are atypical, and most patients are treated for palpable subcutaneous masses with possible symptoms such as pain and limb swelling when the lesion blocks the lumen or when secondary thrombosis occurs. It is difficult to distinguish the disease from venous thrombosis, superficial phlebitis, and other subcutaneous masses. However, the combination of ultrasonography or other auxiliary examinations can improve diagnostic accuracy (44).

On account of the advantages of its simple and non-invasive nature and low cost, ultrasound is the most frequently used auxiliary examination method for finding lower limb masses. With the development and wide application of diagnostic imaging technology, non-invasive ultrasound has been able to provide surgeons with more useful information before surgery. It can clearly show the extent of involvement and the internal blood supply of the lesions, which are helpful for differential diagnosis. The typical sonographic feature of GSV-LMS is a heterogeneous hypoechoic mass in the GSV with a regular or irregular shape that cannot be flattened after compression by a probe. Sometimes, it is difficult to distinguish and easy to be misdiagnosed. When venous thrombosis with an unknown cause or poor therapeutic effect is encountered in clinical work, it is necessary to be vigilant by expanding the scope of the ultrasound scan and carefully observing the relationship between the hypoechoic mass in the venous channel and the venous wall and the internal blood supply of the hypoechoic mass. Ultrasound-guided puncture biopsy can result in a definite diagnosis preoperatively, but due to the unclear origin of the lesion and the possibility of thrombosis, the results may be false negative (47). Contrast-enhanced ultrasound (CEUS) can clearly show the blood supply in a hypoechoic mass, and needle biopsy combined with CEUS can improve the accuracy of diagnosis. Regrettably, due to the patient’s strong willingness to undergo surgery, CEUS, puncture biopsy, and other preoperative examinations such as computed tomography (CT) or magnetic resonance imaging (MRI) were not completed. Therefore, we failed to obtain other images of the lesion.

GSV-LMS needs to be differentiated from other venous diseases. When a venous aneurysm is complicated by thrombosis (56), two-dimensional ultrasound also reveals focal aneurysmal dilation of the GSV with the lumen filled with a hypoechoic material. However, CDFI showed no blood flow signal within the hypoechoic area of the venous aneurysm, whereas abundant blood flow signals can be detected within the aneurysmal lumen of GSV-LMS. Other venous tumors (57–59), such as venous leiomyoma, may exhibit intraluminal growth leading to luminal occlusion or be complicated by venous thrombosis. Compared to GSV-LMS, venous tumors generally demonstrate a slower growth rate. Conventional ultrasound examination poses challenges in differential diagnosis due to overlapping features. Ultrasound-guided needle biopsy can be employed to determine the pathological characteristics, thereby aiding in the formulation of an appropriate treatment plan.

At present, radical resection is the most effective treatment for GSV-LMS. The scope of surgical resection is usually extended to the proximal and distal segments of the affected venous margin of approximately 2–3 cm so as to reduce local recurrence and minimize the negative margin. When there is obvious adhesion between the lesion and the surrounding tissues, the scope of resection can be further expanded according to the actual situation, and vascular reconstruction or autologous vein transplantation can be performed when necessary (1). Only three of the reported cases underwent intraoperative vascular reconstruction. Preoperative ultrasound evaluation can help to identify the nature of the lesion, evaluate the scope of lesion involvement, and guide the formulation of a surgical plan, which can reduce partly the recurrence rate of patients.

Currently, the indications and efficacy of adjuvant chemoradiotherapy for venous LMS are unclear. Postoperative adjuvant chemoradiation is often performed for high-grade tumors clinically, which can effectively prevent local recurrence and metastasis after surgery. A total of 17 cases in this study received adjuvant treatment after surgery, and none of these cases had local recurrence or metastasis during the reported postoperative follow-up period. Ultrasound is of great value in evaluating whether patients have recurrence or metastasis and can effectively assist in judging patients’ condition to aid in guiding chemoradiotherapy. Patients with GSV-LMS have a high probability of tumor recurrence within 2 to 3 years after surgery, so patients should have regular postoperative follow-ups. Evaluations such as ultrasound every 3 months and other imaging examinations such as CT every 6 months are usually recommended (48).

GSV-LMS is an extremely rare malignant tumor originating from veins, which is difficult to diagnose and prone to misdiagnosis and mistreatment. Preoperative ultrasound examination has a certain evaluation value. It can assist in guiding clinical diagnosis and treatment and in improving the diagnosis and treatment effect. GSV-LMS patients may have recurrence and metastasis, and ultrasound can be used as the most convenient follow-up tool after surgery to detect new lesions in time, which is of great value in improving the long-term survival rate of patients.

## Data Availability

The raw data supporting the conclusions of this article will be made available by the authors, without undue reservation.
